# Spontaneously Resolved Penile Cyst in a Fetus: A Case Report and Review of the Literature

**DOI:** 10.7759/cureus.28110

**Published:** 2022-08-17

**Authors:** Arturan Ibrahimli, Kamran Huseynli, Aysuna Galandarova, Elgun Samadov, Arzu Jafarova

**Affiliations:** 1 Medicine, Ankara University School of Medicine, Ankara, TUR; 2 Plastic and Reconstructive Surgery, Ankara Yıldırım Beyazıt University, Ankara, TUR; 3 Surgery, Leyla Medical Center, Baku, AZE; 4 Radiology, Shafa Hospital, Baku, AZE

**Keywords:** fetal, imaging, ultrasound, cyst, penile

## Abstract

Cysts are uncommon congenital lesions of the fetal penis. Fetal penile cysts can develop when epithelial cells become entrapped during the fusion of the labial scrotal folds. The spectrum of diagnoses varies from simple epidermal inclusion cysts and megalourethra to hypospadias. In our case, we present a penile cyst that appeared between the 16^th^ and 24^th^ weeks. There was no other congenital anomaly, and since the mother did not accept an amniocentesis, we could not learn the karyotyping findings. As there were no other signs of congenital anomalies on ultrasound, we decided only to follow up, and at the 24^th^ week’s control ultrasound, the cyst was completely resolved with no other imaging findings.

## Introduction

Cysts in the penis are rare lesions and can occur anywhere from the urethral meatus to the base of the penis [1]. Fetal penile cysts can develop when epithelial cells become entrapped during the fusion of the labial scrotal folds. The spectrum of diagnoses varies from simple epidermal inclusion cysts and megalourethra to hypospadias. The majority of penile cystic lesions mentioned in the literature are median raphe cysts [1,2].^ ^These are also known as para-meatal cysts when they are close to the meatus [3]. We present a case of a penile cyst that appeared at the 16^th^ week of pregnancy and resolved spontaneously by the 24^th^ week. This rare case will be a source for clinicians who are looking for information about the management of penile cysts presenting with no other congenital anomaly.

## Case presentation

**Figure 1:**
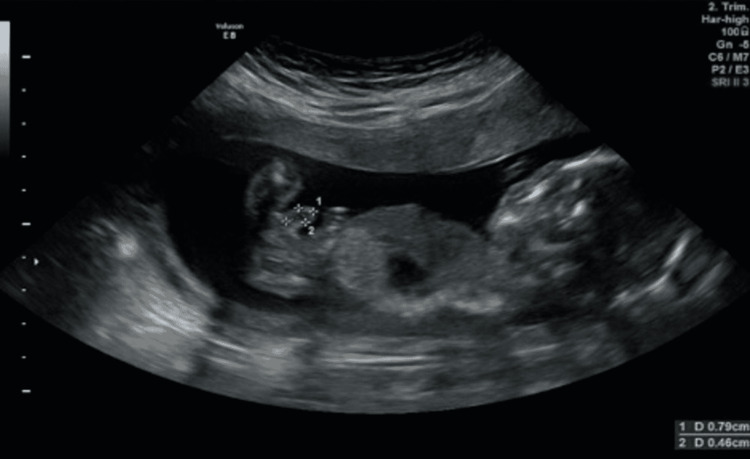
16th week ultrasound

**Figure 2:**
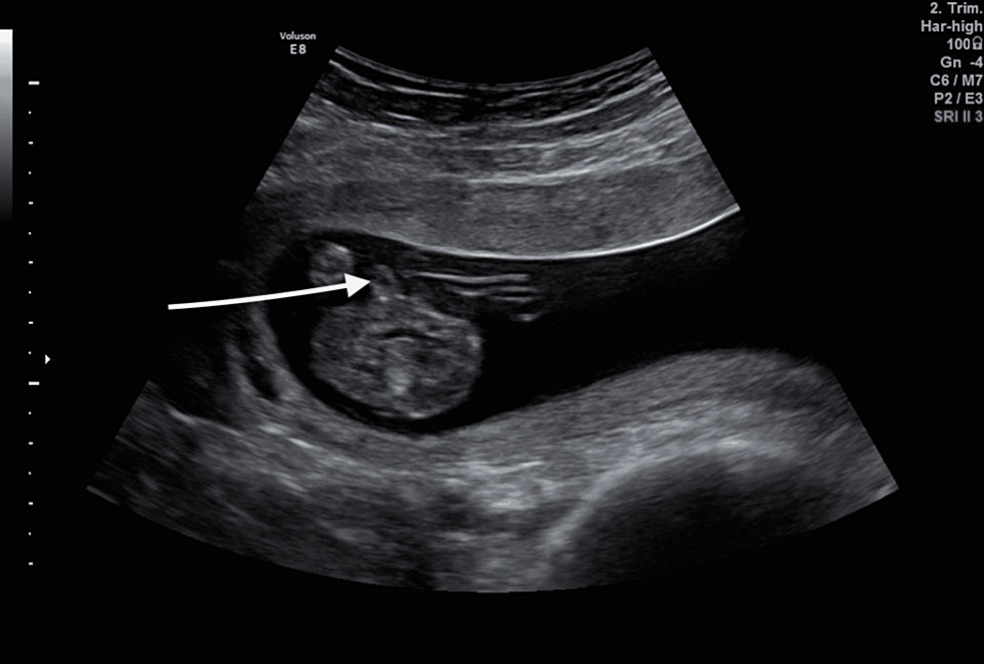
16th week ultrasound

In our case, a 35-year-old mother, gravida 4, paravida 3, came to our clinic for a routine 16^th^ week ultrasound control. All her previous pregnancies were uncomplicated, two of them with vaginal delivery and the last one with cesarean section. During the examination, a cyst with the dimensions of 0.79x0.49 cm was noticed in the midline of the penis at the 16^th^ week control ultrasound (Figure 1, 2).

**Figure 3:**
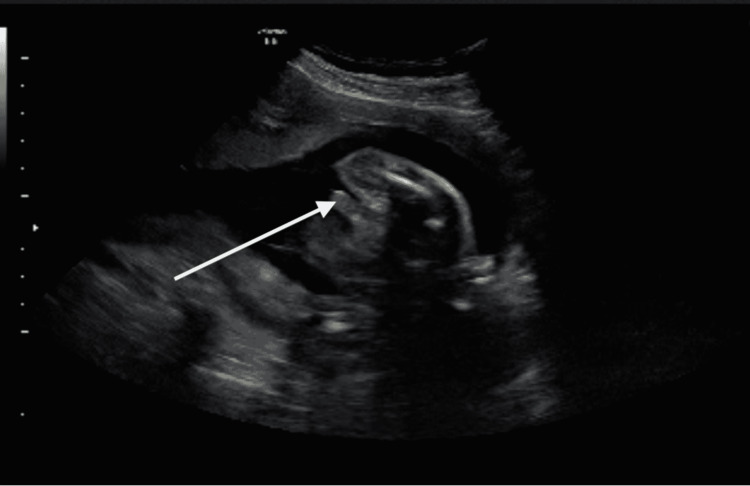
24th week ultrasound The cyst is completely resorbed

At the 12^th^ week follow-up, there were no abnormalities of the penis. The mother refused an amniocentesis; therefore, karyotype analysis could not be performed. No other congenital anomaly was detected during the examination. Since karyotype analysis could not be performed and there was no other accompanying anomaly, we decided to continue the pregnancy with close follow-ups. The cyst was still visible at the 20^th^ week, but it resolved spontaneously by the 24^th^ week (Figure 3). The 35-year-old mother had her fourth birth with a cesarean section. A healthy baby was born with a weight of 2810 grams. The boy was born with an anatomically and functionally normal penis without findings of hypospadias or cyst. The child was healthy, and no postpartum pathological findings were detected.

## Discussion

Cysts are rare, benign, typically asymptomatic lesions of the penis. They are classified as congenital cysts and acquired cysts. Congenital dermoid cysts, median raphe cysts, parameatal cysts, and mucoid cysts are examples of congenital penile cysts [3,4].

The first thought was that the anomaly detected in the patient might be anterior urethral diverticulum, median raphe cyst, urethral cyst, inclusion cyst, hypospadias, or megalourethra.

Anterior urethral diverticulum (AUD) is a rare congenital defect in children that cause lower urinary tract blockage. Difficulty in voiding, dribbling during micturition, a poor urinary stream, or recurrent UTI are all common presenting complaints [5,6]. The size of the diverticulum and the degree of obstruction determine the treatment for AUD. For tiny, well-supported diverticula, transurethral resection with a pediatric resectoscope is the therapy of choice. Open diverticulectomy and primary repair are advisable in big diverticula [7,8].

A median raphe cyst is a rare, mostly asymptomatic condition that may appear along the midline from the glans to anus. There are two different approaches, such as excision or observation if it is small and asymptomatic [2]. These cysts most frequently develop in the ventral penis and in the parameatal position [9]. Parameatal urethral cysts are uncommon benign congenital penis lesions firstly described by Lantin and Thompson in 1956 in literature [10]. They may be treated by surgical excision if they do not resolve on their own and cause urination problems or for cosmetic concerns.

Hypospadias is one of the most frequent congenital genital defects in males. A proximal displacement of the urethral opening, penile curvature, and a ventrally lacking hooded foreskin are all common symptoms [11]. Further diagnostic assessment, such as ultrasonography of the urinary tract and internal genital organs to detect additional nephrourological anomalies, is recommended in proximal and complicated hypospadias. The ideal time for primary surgical repair is at six to 12 months of life, although when this is not practicable, there is another opportunity at three to four years old [12].

Congenital megalourethra is an uncommon urogenital abnormality characterized by penile urethral dilation and elongation, as well as the absence or hypoplasia of the corpora spongiosa and cavernosa. Urinary and erectile dysfunction, renal insufficiency, and pulmonary hypoplasia are all postnatal complications [13]. Due to its relationship with obstruction, which is not present in this case, the likelihood of megalourethra is low.

Since penile anomalies carry risks such as sexual problems, urination problems, and cosmetic problems, they should be followed closely and surgically corrected if necessary. Surgery indications at penile cysts are urinary tract obstruction, secondary infection, pain, or cosmetic considerations [14]. However, the cyst we observed, in this case, disappeared spontaneously until delivery without any additional procedure, and no anomaly was detected in the child after birth. The lack of similar cases in the literature and the lack of detailed guidelines and recommendations are the reasons that complicate the decision to follow up on such cases. 

## Conclusions

To summarize, fetal penile cysts are uncommon ultrasound findings that can be associated with significant malformations. However, in cases where there are no other accompanying anomalies proven after detailed examination and karyotype analysis is normal, it may be the best option to follow up.
